# Unexpected Paraganglioma Identified During Bilateral Nephrectomy in Autosomal Dominant Polycystic Kidney Disease: A Case Report

**DOI:** 10.7759/cureus.92464

**Published:** 2025-09-16

**Authors:** Mehdi Vollet, Catherine Blanc, Maurice Matter, Gregory Hofmann

**Affiliations:** 1 Anesthesiology, Lausanne University Hospital (CHUV) and University of Lausanne, Lausanne, CHE; 2 Visceral Surgery, Lausanne University Hospital (CHUV) and University of Lausanne, Lausanne, CHE

**Keywords:** autosomal dominant polycystic kidney disease, hypertension, nephrectomy, paraganglioma, perioperative management

## Abstract

Autosomal dominant polycystic kidney disease (ADPKD) is commonly associated with hypertension, which is typically attributed to renal dysfunction. However, secondary causes of hypertension, particularly pheochromocytomas and paragangliomas (PPGLs), should be considered in cases of severe or difficult-to-control hypertension.

We report the case of a 77-year-old woman with ADPKD and end-stage renal disease who underwent bilateral nephrectomy before kidney transplantation. Despite requiring four antihypertensive medications, her hypertension was attributed to renal disease. During left nephrectomy, manipulation of a perirenal mass resulted in a severe hypertensive crisis followed by refractory hypotension. Intraoperative frozen section analysis revealed an unsuspected 6.3-cm paraganglioma, while final pathology further demonstrated a 13-cm perirenal metastasis of the previously known myxoid liposarcoma, and also identified a second 0.4-cm peri-aortic paraganglioma.

The patient experienced significant perioperative hemodynamic instability but recovered without organ failure. Postoperative complications included arteriovenous fistula occlusion, which was successfully managed. The discovery of metastatic disease led to reassessment of transplant candidacy.

This case highlights the importance of considering PPGLs in ADPKD patients with severe hypertension requiring multiple antihypertensive medications. Preoperative biochemical screening and appropriate perioperative management are crucial for patient safety. The complexity of imaging interpretation in ADPKD patients requires careful multidisciplinary evaluation to exclude neoplastic processes.

## Introduction

Autosomal dominant polycystic kidney disease (ADPKD) is the most common form of polycystic kidney disease, affecting 1 in 400 to 1,000 births. The condition results from mutations in the PKD1 gene in approximately 85% of cases and the PKD2 gene in about 15% of cases, making it the most common genetic disorder leading to end-stage renal disease by age 70 [1]. Hypertension is a prominent feature of ADPKD, affecting 50% of patients between ages 20 and 34 with preserved renal function and nearly 100% of patients with end-stage renal disease [1]. While hypertension in ADPKD is typically attributed to renal dysfunction, secondary causes must be considered, particularly when blood pressure control is difficult despite multiple antihypertensive medications. Pheochromocytomas and paragangliomas (PPGLs) are rare neuroendocrine tumors that can cause severe hypertension and life-threatening hemodynamic instability. Although only a few cases have reported the coexistence of ADPKD and PPGLs [2-5], the recognition of this association is crucial for appropriate perioperative management and patient safety. We present a case of incidental paraganglioma discovery during bilateral nephrectomy in a patient with ADPKD, highlighting the importance of considering secondary causes of hypertension and the challenges of imaging interpretation in this population.

## Case presentation

**Figure 1:**
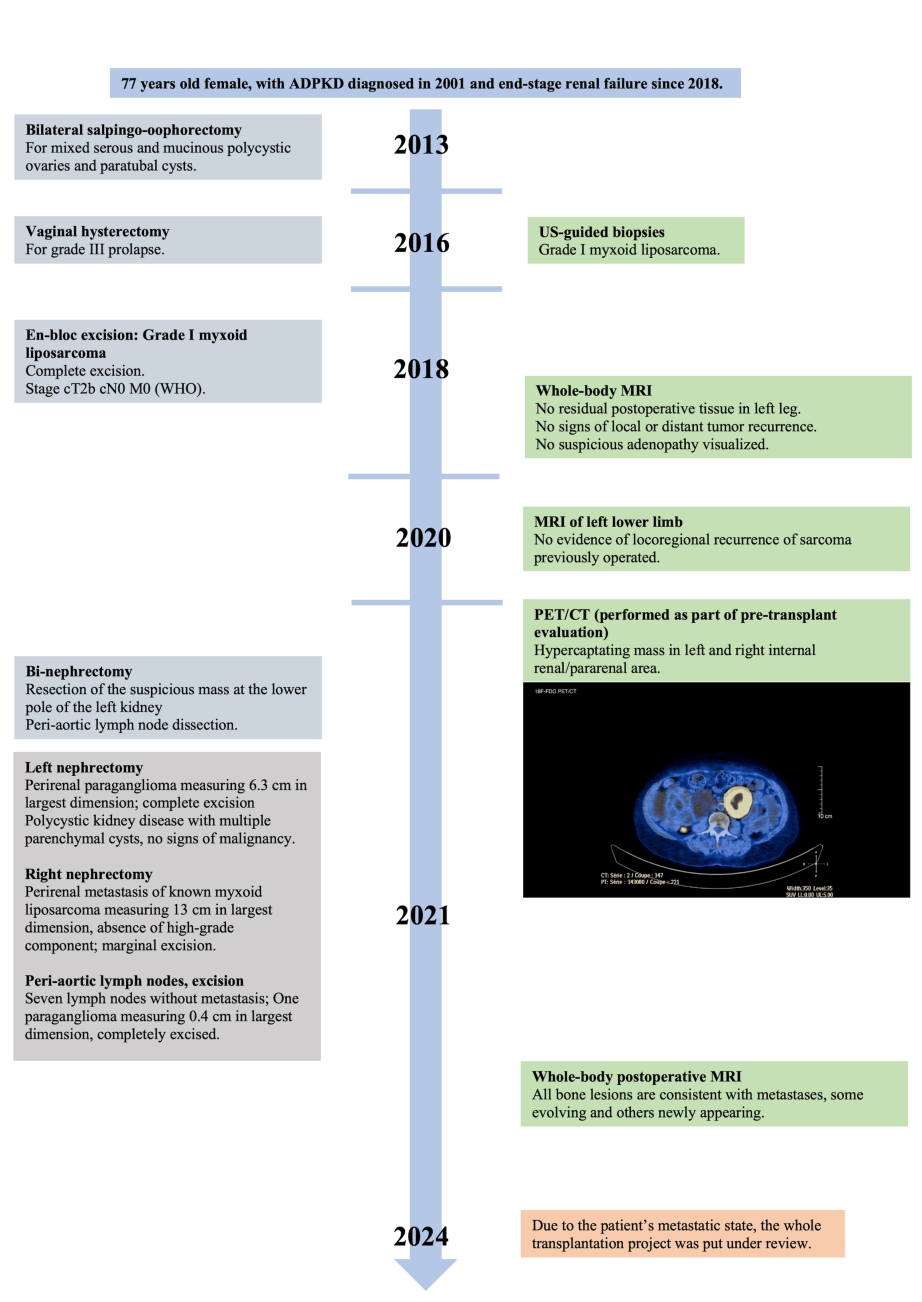
Timeline of clinical events organized according to CARE guidelines. Image credit: Mehdi Vollet.

A 77-year-old woman with ADPKD and end-stage renal disease was scheduled for bilateral nephrectomy before kidney transplantation due to excessive kidney size. Her medical history (Figure 1) included stage I myxoid liposarcoma of the left leg resected four years earlier, permanent pacemaker placement for atrioventricular block, and hypertension requiring four antihypertensive medications.

**Figure 2:**
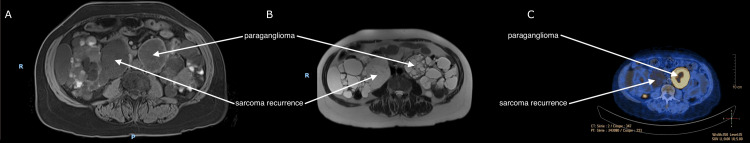
Preoperative imaging studies. (A) T1-weighted magnetic resonance imaging (MRI) sequence showing heterogeneous cysts consistent with ADPKD bilaterally. (B) T2-weighted MRI sequence demonstrating stable cystic lesions. (C) PET-CT scan showing a hyperactive left-sided cyst initially interpreted as infectious. ADPKD, autosomal dominant polycystic kidney disease; PET-CT, positron emission tomography-computed tomography

Annual surveillance imaging for her previous sarcoma was systematically reviewed by tumor board specialists. An 18-fluorodeoxyglucose positron emission tomography/computed tomography (18F-FDG PET-CT) scan performed to exclude neoplastic recurrence revealed a 6.0-cm right internal pararenal lesion and a 6.2-cm left internal pararenal lesion with necrotic centers. Complementary magnetic resonance imaging demonstrated stable heterogeneous lesions bilaterally with no suspicious features. All imaging was reviewed by local radiologists and the dedicated sarcoma tumor board radiologist at the tertiary reference center (Figure 2). 

Antihypertensive therapy was intensified following the preoperative anesthesia consultation one week before admission. Hemodialysis was performed the day before surgery. Preoperative laboratory values are presented in Table 1. 

**Table 1:** Preoperative laboratory values. eGFR, estimated glomerular filtration rate

	Value	Reference range
eGFR (mL/minute/1.73 m^2^)	8	>60
Serum creatinine (mmol/L)	455	44-80
Serum sodium (mmol/L)	140	135-145
Serum potassium (mmol/L)	4.9	3.5-4.6
Hemoglobin (g/L)	123	117-157

Perioperative management and intraoperative events

Upon arrival in the operating room, the patient was hypertensive (185/60 mmHg). Epidural catheter placement required multiple attempts, during which the patient received 1 mg midazolam. Anesthesia was induced with propofol, fentanyl, and cisatracurium. Tracheal intubation was successful on the first attempt. Anesthesia was maintained with sevoflurane, and bispectral index monitoring was used to assess anesthetic depth.

Shortly after surgical incision, the patient developed an acute hypertensive crisis with a peak blood pressure of 210/95 mmHg, requiring administration of 0.5 mg nicardipine. During manipulation of a 5-cm left pararenal mass, the patient experienced a second severe hypertensive crisis reaching 210/80 mmHg. This episode was unresponsive to epidural bupivacaine titration and required nicardipine administration along with increased sevoflurane concentration.

Given the temporal relationship between mass manipulation and hemodynamic instability, a paraganglioma was suspected. The left nephrectomy was expedited, immediately followed by severe refractory hypotension requiring norepinephrine up to 35 μg/minute. Mean arterial pressure remained below 60 mmHg despite the addition of phenylephrine and epinephrine (up to 40 μg bolus) along with aggressive fluid resuscitation. Blood pressure normalized after 40 minutes while maintaining high-dose vasopressor support.

**Figure 3:**
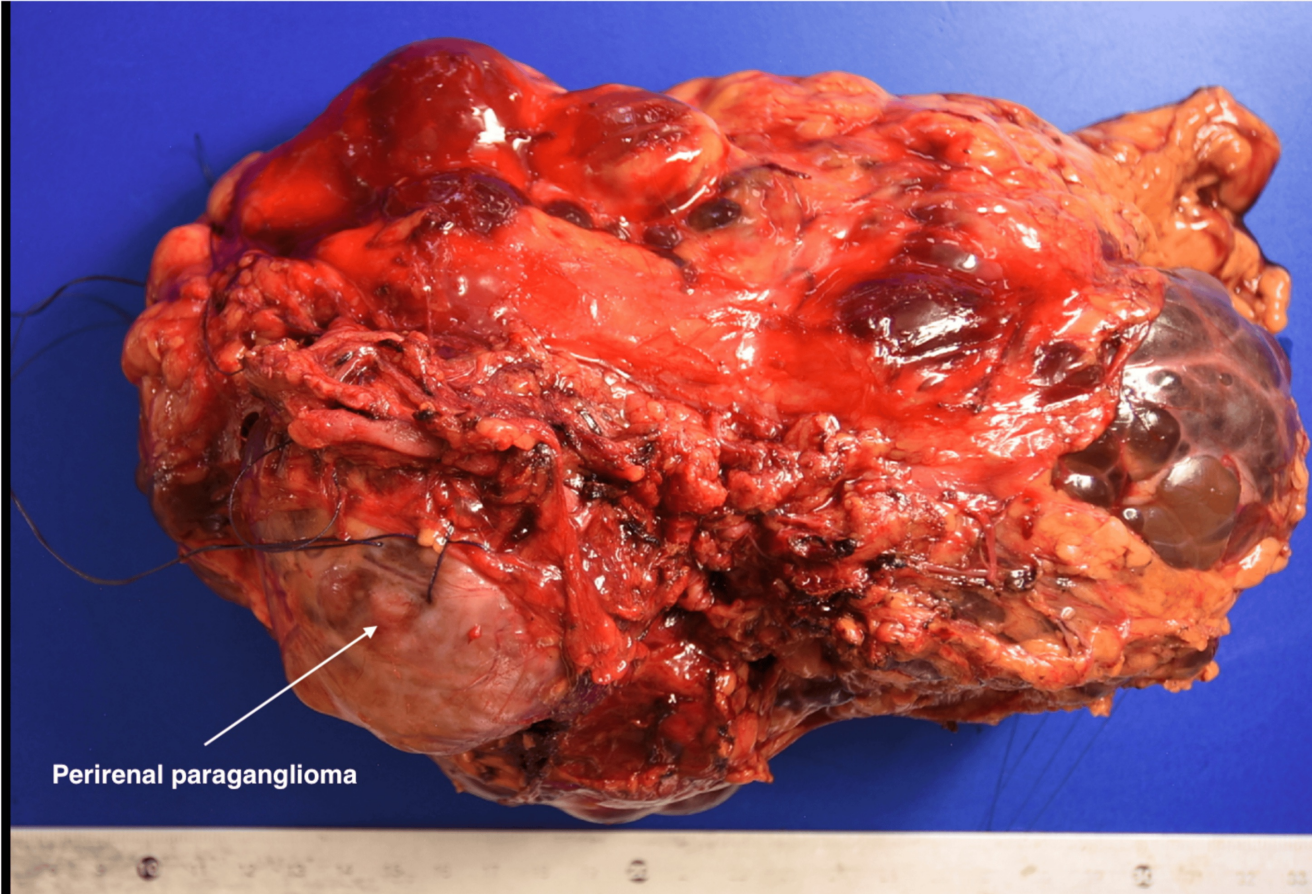
Left nephrectomy specimen showing a perirenal paraganglioma measuring 6.3 cm in its largest dimension, completely excised. The kidney exhibits polycystic disease with multiple parenchymal cysts and no evidence of malignancy.

Due to the hemodynamic instability, intraoperative frozen section analysis was performed (Figure 3), confirming the presence of an unsuspected perirenal paraganglioma. Intra-aortic and caval lymph node dissection was performed for histopathological sampling. The right nephrectomy proceeded without complication. Estimated blood loss was 700 mL. The patient was transferred to the intensive care unit, sedated, and mechanically ventilated.

Postoperative course and outcomes

The patient experienced no organ failure during her intensive care unit stay. Vasopressor support was successfully weaned on postoperative day 1, and the patient was extubated without complication. On postoperative day 7, she developed arteriovenous fistula occlusion, which was successfully managed with surgical thrombectomy under local anesthesia and propofol sedation without complications.

No other postoperative complications occurred. The patient remained in the visceral surgery department for an additional 22 days before discharge home to continue thrice-weekly hemodialysis. Final histopathological examination revealed a 13-cm metastatic myxoid liposarcoma on the right side. Lymph node dissection showed seven negative nodes and identified a second 0.4-cm paraganglioma.

The patient was discharged on postoperative day 25 with scheduled follow-up appointments in nephrology and oncology clinics. Due to the metastatic disease, kidney transplantation candidacy was reassessed.

## Discussion

PPGLs are uncommon neuroendocrine tumors originating from chromaffin cells of the autonomic nervous system. Pheochromocytomas arise from the adrenal medulla, while paragangliomas develop from extra-adrenal chromaffin tissue. The 2022 World Health Organization Classification of Endocrine and Neuroendocrine Tumors now classifies pheochromocytomas as intra-adrenal paragangliomas, reflecting their shared origin and behavior [6].

PPGLs demonstrate one of the highest hereditary rates among solid tumors, with approximately 40% being hereditary and more than 20 genes implicated in their pathogenesis. Some genes are associated with syndromic forms (RET, VHL, EPAS1, NF1, FH) [7]. Current genetic classification divides PPGLs into three clusters: cluster 1 includes genes related to the Krebs cycle and hypoxia signaling pathway; cluster 2 encompasses genes related to kinase signaling pathways; and cluster 3 includes CSDE1 and UBTF-MAML3 genes [8].

The annual prevalence of PPGLs ranges from 0.04 to 0.95 cases per 100,000 people, with prevalence among hypertensive patients varying from 0.2% to 0.6% [9,10]. Recent meta-analyses suggest that classic symptoms are less common than previously reported, with headaches occurring in 60% of patients, palpitations in 60%, and diaphoresis in 52% [11]. Hypertension remains the most prevalent symptom, affecting up to 95% of patients, with 75% experiencing severe hypertension and 7% to 17% developing catecholamine-induced hypertensive crises [12].

Diagnosis of PPGLs requires a combination of biochemical testing and imaging studies. Initial biochemical evaluation should include measurement of plasma-free metanephrines or 24-hour urinary fractionated metanephrines. Once biochemical evidence is established, computed tomography is the preferred imaging modality for localization. Magnetic resonance imaging serves as a secondary option when CT results are inconclusive or when patients are unsuitable for contrast-enhanced imaging. Functional imaging is recommended for patients at high risk for metastatic or multifocal disease [8].

Surgical resection remains the only potentially curative treatment for PPGLs. The primary goals of surgery are to eliminate risks associated with excessive hormone secretion and prevent tumor progression. The effects of catecholamine excess must be carefully managed through medical therapy before and during surgical procedures [13].

Perioperative management of PPGL patients requires meticulous attention to hemodynamic stability. All patients should receive preoperative alpha-adrenergic blockade to control catecholamine excess symptoms and blood pressure. Betablockers should be avoided until alpha-blockade is established. Additional recommendations include high-sodium diet during alpha-blocker therapy and saline infusion 24 hours before surgery [11].

Intraoperative hypertensive crises should be managed with adequate anesthetic depth and short-acting parenteral antihypertensive agents, including nitric oxide modulators, calcium channel blockers, alpha-adrenergic antagonists, and magnesium sulfate. Tachyarrhythmias can be treated with beta-adrenergic antagonists. Recent studies suggest that alpha-2-adrenergic agonists like dexmedetomidine may be effective in reducing catecholamine availability [14]. However, these studies excluded patients with significant cardiac, renal, and hepatic comorbidities, limiting generalizability and highlighting the need for further research. Following tumor isolation, severe refractory hypotension may require vasopressor agents combined with aggressive fluid resuscitation [15].

Postoperative complications primarily include hemodynamic instability and hypoglycemia [11]. Hypotension typically results from rapid decline in circulating catecholamines after tumor removal, residual effects of preoperative antihypertensive medications, hypovolemia, blood loss, or adrenoreceptor downregulation. Hypoglycemia usually occurs within hours of tumor removal due to increased insulin secretion following catecholamine decrease [16].

This case demonstrates the complexity of managing patients with ADPKD and severe hypertension. Despite the patient's long history of hypertension attributed to renal disease, the perioperative hemodynamic instability clearly indicated catecholamine excess. The initial imaging interpretation failed to recognize the paraganglioma, highlighting the challenges of distinguishing between cystic lesions and solid masses in patients with ADPKD. Literature review identified only four similar cases of coexistent ADPKD and PPGLs, with no established causal relationship between these conditions [2-5]. When evaluating hypertension in patients with ADPKD, clinicians must consider differential diagnoses beyond renal failure, particularly when multiple antihypertensive medications are required for blood pressure control.

Key clinical messages

Key clinical lessons from this case include:

(1) Think beyond the kidney: In patients with ADPKD and difficult-to-control hypertension, secondary etiologies like PPGLs should be actively considered, even in the absence of classic catecholaminergic symptoms.

(2) Imaging can be deceptive: The complex anatomy of polycystic kidneys can obscure solid masses on conventional imaging. Multidisciplinary review of imaging studies, supplemented when appropriate by functional imaging, is critical to prevent missed diagnoses.

(3) Preparation prevents peril: The intraoperative discovery of an unprepared PPGL exposes patients to extreme hemodynamic risk. A preoperative diagnosis is, therefore, essential, as it allows for proper medical optimization, which is a fundamental principle of safe surgical management.

## Conclusions

This case illustrates the importance of systematically investigating alternative etiologies of hypertension in patients with ADPKD, particularly when blood pressure control is difficult despite multiple medications. The complexity of imaging interpretation in patients with ADPKD requires careful multidisciplinary evaluation to exclude neoplastic processes, including PPGLs and renal cell carcinoma.

Integration of molecular imaging modalities and biochemical analysis should be considered to effectively exclude PPGLs as contributory factors in hypertension, especially in patients being evaluated for kidney transplantation or undergoing major surgical procedures. Thorough preoperative assessment and appropriate perioperative management strategies are essential for patient safety and optimal outcomes.

Future research should focus on establishing screening protocols for secondary causes of hypertension in patients with ADPKD and developing improved imaging techniques for distinguishing between cystic lesions and solid masses in this population.
